# Assessment of knowledge, attitude, and practice regarding the disposal of expired and unused medications among the Lebanese population

**DOI:** 10.1186/s40545-022-00506-z

**Published:** 2022-12-30

**Authors:** Aline Hajj, Souraya Domiati, Chadia Haddad, Hala Sacre, Maria Akl, Marwan Akel, Samah Tawil, Soula Abramian, Rony M. Zeenny, Fadi Hodeib, Pascale Salameh

**Affiliations:** 1 Faculty of Pharmacy, Université Laval, Québec City, Canada; 2 INSPECT-LB (Institut National de Santé Publique, d’Épidémiologie Clinique et de Toxicologie-Liban), Beirut, Lebanon; 3 Oncology Division, CHU de Québec-Université Laval Research Center, Québec City, QC Canada; 4 Laboratoire de Pharmacologie, Pharmacie Clinique et Contrôle de Qualité Des Médicaments (LPCQM), Faculty of Pharmacy, Saint-Joseph University of Beirut, Beirut, Lebanon; 5 Department of Pharmacology and Therapeutics, Faculty of Pharmacy, Beirut Arab University, Beirut, Lebanon; 6 School of Medicine, Lebanese American University, Byblos, Lebanon; 7 Research Department, Psychiatric Hospital of the Cross, Jal Eddib, Lebanon; 8 School of Health Sciences, Modern University for Business and Science, Beirut, Lebanon; 9 Drug Information Center, Order of Pharmacists of Lebanon, Beirut, Lebanon; 10 Faculty of Public Health and Policy, London School of Hygiene & Tropical Medicine, London, UK; 11 Pharmacy Practice Department, School of Pharmacy, Lebanese International University, Beirut, Lebanon; 12 International Pharmaceutical Federation, The Hague, Netherlands; 13 Department of Pharmacy, American University of Beirut Medical Center, Beirut, Lebanon; 14 Department of Biomedical Sciences, School of Pharmacy, Lebanese International University, Beirut, Lebanon; 15 Department of Primary Care and Population Health, University of Nicosia Medical School, 2417 Nicosia, Cyprus; 16 Faculty of Pharmacy, Lebanese University, Hadat, Lebanon

**Keywords:** Medication waste, Expiry, Environment, Garbage stream, Community, Pharmacy

## Abstract

**Background:**

Medication waste is a public health problem affecting developed and developing countries. In Lebanon, a developing country in the Middle East, efforts are being deployed in hospitals but not in the community.

**Objective:**

This study aimed to validate a questionnaire to explore the knowledge, attitude, and practice (KAP) towards the disposal of unused and expired medicines among the Lebanese population and then identify the factors associated with these variables comparatively between the general population and healthcare professionals.

**Methods:**

A cross-sectional study was conducted among the general Lebanese population in May–June 2022 using a standardized questionnaire. The validity and reliability of the KAP scales were assessed, then a thorough statistical analysis was done to explore the factors associated with these scales.

**Results:**

The KAP scales generated by this study were valid and reliable. Using these scales, 24.5%, 22.6%, and 21% of participants demonstrated proper knowledge, attitude, and practice, respectively. Higher knowledge scores were significantly associated with female gender (Beta = 0.97), a high monthly income (Beta = 1.68), a secondary (Beta = 6.11) or university (Beta = 6.80) education level, and postgraduate education (Beta = 7.13). However, older age (Beta = − 0.06) and a low monthly income (Beta = − 3.06) were significantly associated with lower knowledge scores. A higher knowledge score (Beta = 0.06) was significantly associated with a more positive attitude regarding unused or expired medication disposal. Being a healthcare professional (Beta = 0.72) was significantly associated with a higher practice score, while being a female (Beta = − 0.32) and living in a rural area (Beta = − 0.37) were significantly associated with lower practice scores.

**Conclusion:**

This study validated KAP scales regarding medication waste in Lebanon and showed low KAP scores in the majority of respondents. Factors associated with higher KAP scores in various aspects of medication disposal, including gender, age, education level, and profession (healthcare professionals), suggest the need to consider those when implementing targeted corrective measures. Although further studies are required to confirm our findings, this study could be the ground for a medication waste management national strategy in Lebanon.

**Supplementary Information:**

The online version contains supplementary material available at 10.1186/s40545-022-00506-z.

## Background

Medications are effective and safe if stored under the appropriate conditions mentioned on the label and used before the expiry date is reached [1]. Manufacturers and some health organizations advise disposing of medications by their expiry date [2], as expired medications may decompose and become ineffective or even dangerous. Further, their rational use is being highlighted, given their growing consumption worldwide. In many instances, prescribed medications remain unused because of treatment changes, side effects, patient health improvements, or other factors [3]. According to the World Health Organization, a large amount of medicine is inappropriately prescribed or sold, resulting in tons of solid waste of expired and unused medications and a medication disposal burden [3].

Inappropriate disposal of medications endangers human health and ecosystems  [4]. Some people keep unwanted, unused, or expired medications in their homes indefinitely, while others dispose of them in general municipal waste bins or sinks, or flush them down the toilet  [4]. Consequently, trace amounts of pharmaceuticals and their metabolites have been identified in some drinking water supplies, exposing humans to the accumulation of these traces through contaminated drinking water [5]. Waterways have also been found to contain antibiotics, antidepressants, and hormone replacement therapy [6]. Inappropriate medication disposal also leads to the potential risk of medication abuse related to narcotic pain relievers and sleep aids and could contribute to antimicrobial resistance [4]. Children and pets could also be at an increased risk of accidental poisoning due to the ease of access to unused, inappropriately discarded, or insecurely left-at-home medications [7].

Several studies have revealed a lack of proper disposal of unused or expired medications, particularly in developing countries. For example, a study in India showed that only 39% of the studied sample were aware of appropriate disposal methods for expired medications [8]. Similarly, the majority of study participants in Turkey disposed of pharmaceutical waste against national and international policies [9]. The situation is almost the same in Kuwait, where 76.5% of the surveyed population threw unwanted drugs in the garbage, and 11.2% flushed them down the drain [10]. A Saudi Arabian study found that 48.1% of participants discarded expired medications in household garbage [11]. In 2022, only 6.5% of Saudis had proper knowledge of expired medication disposal [12]. Other studies from around the world, including developed countries with guidelines and regulations, have revealed that disposal via the solid waste garbage stream, sink, and toilet is common practice [13–22]. In Lebanon, a study among residents in the administrative area of the capital city, Beirut, showed that 78.3% of respondents disposed of unwanted solid-form medications in garbage, 6% flushed them down the toilet or sink, 8.5% gave them to dispensaries or people in need, and 3.6% returned them to the pharmacy. The percentages were similar for liquid medications [23]. This improper disposal of expired medications has detrimental effects on the environment, especially with the increased hoarding in the era of COVID-19 [24]. Furthermore, many countries lack regulations and programs for the appropriate management of medication waste [25].

Besides their harmful effects on ecosystems and health, medication waste products also have unfavorable economic consequences, straining healthcare systems globally. In 2012, medication waste and suboptimal use of medicines accounted for around £300 million and more than $418 billion in England and the United States (US), respectively [26]. A study from 2003 found that families in Saudi Arabia and other Gulf countries spent a total of around $150 million on unused medicines [27].

Based on the above, waste prevention strategies are warranted at all levels (prescriber, dispenser, and patient) to reduce the economic and environmental burdens of medication waste [27] and align with the United Nations’ Sustainable Development Goals [28], mainly good health and well-being (SDG3), clean water and sanitation (SDG6), sustainable cities and communities (SDG11), and life on land (SDG15), and the International Pharmaceutical Federation’s Development Goals [29], including patient safety (DG19) and sustainability in pharmacy (DG21). Patients are encouraged to make savvy purchases, buying instead of hoarding the medicines they need, and pharmacists can play an essential role in reducing their stock, educating patients about waste, and dispensing medications judiciously [7, 27]. Safer disposal methods should also be adopted, similar to those in developed countries.

In the US, the Food and Drug Administration (FDA) issued guidelines to consumers on where and how to dispose of unused medicines, stating that the best way to dispose of unused or expired medications is to place them in medication take-back sites, which may be located in pharmacies, hospitals, or other healthcare settings. Otherwise, medications can be flushed down the toilet if listed on the FDA flush list, including opioids (buprenorphine, fentanyl, hydrocodone, meperidine, morphine, and others), sodium oxybate, diazepam, and methylphenidate. In contrast, non-flush list medications should be mixed with an unappealing substance such as dirt, cat litter, or used coffee grounds, placed in a sealed plastic bag, then thrown in the trash [22]. In Dubai, the Ministry of Health and Prevention launched an e-service to dispose of unused or expired drugs in 2020 [30].

In Lebanon, some efforts are being made in this regard, but the system is still not functioning adequately. The Ministry of Public Health (MOPH) has issued several decrees in the past years to regulate healthcare waste generated by hospitals, not those produced by the general population, following various laws and international conventions [31]. In community pharmacies, suppliers either return money or replace near-expiry goods with newer ones six to twelve months before they expire (it is of note that due to the current socioeconomic and sanitary crises, this procedure is not functioning correctly, and pharmacists are not able to return any expired medications to suppliers). At any point beyond this moment, pharmacies are no longer liable for the consequences of improperly discarding medicines or how this might affect the environment; expired drugs fall then into the hands of the Lebanese Pharmaceutical Importers and Wholesalers Association (LPIA), which bears the responsibility for destroying medications in local incinerators when available or exporting them to be destroyed abroad [32].

In light of this context, with the increasing consumption of medicinal products, the inappropriate disposal of unused and expired medications could cause environmental harm and public health problems. Exploring the current knowledge, attitude, and practices towards medication disposal of the general population provides insight into this matter and helps minimize medication waste, implement efficacious measures and proper disposal guidelines, and improve public awareness [33]. Since the only previous Lebanese study [23] targeted a sample from the administrative region of Beirut only and did not use validated tools to assess the disposal of pharmaceuticals in Lebanese households, it was deemed necessary to explore the knowledge, attitude, and practices (KAP) of the general population and healthcare professionals regarding the various aspects of medication disposal to help develop new strategies to improve the use and disposal of medications. Moreover, since KAP aspects would be better assessed using a valid and reliable instrument, such an instrument would also allow the determination of the facilitators and barriers to proper practice [34]. Therefore, this study aimed to validate a questionnaire to explore KAP towards the disposal of unused and expired medicines among the Lebanese population and then identify the factors associated with these variables comparatively between the general population and healthcare professionals.

## Material and methods

### Study design

An observational cross-sectional study was conducted in May–June 2022 among 415 Lebanese adults (including non-healthcare and healthcare professionals). Participants were recruited from all Lebanese districts using a snowball sampling technique and received no incentive for their participation, which was voluntary.

### Sample size

In the absence of similar studies in Lebanon, the Epi-Info 7 software was used to calculate a minimum sample of 384 participants, assuming that the percentage of participants who can be considered knowledgeable about medication disposal is 50%, with an acceptable margin of error of 5%. Accordingly, the survey portal was closed when the number of responses exceeded the minimum required.

### Data collection

An online questionnaire in Arabic and English (Additional file 1) was created on Google Forms (https://forms.gle/bQNTGRfeFWLgUL8b6). The link to the questionnaire was shared via WhatsApp and other social media platforms. Participants were also encouraged to disseminate the survey to others using the snowball technique, thus reaching people from all Lebanese districts.

The study scope and purpose were explained at the beginning of the questionnaire. Participants were informed that their participation in the study was voluntary, and they were assured that their responses would remain anonymous and confidential. The Institutional Review Board at Beirut Arab University approved the study.

### Data collection tool

The 40-item questionnaire included two main sections: sociodemographics and knowledge, attitudes, and practices related to the disposal of unused and expired medications. Age, gender, marital status, level of education, region, career, household income, household crowding, and the presence of a healthcare professional in the family were all covered in the sociodemographic part.

Questions related to waste KAP were retrieved from several articles and modified to fit the purpose of the study after a thorough review of the literature [8, 9, 35–38]. The questions were reviewed by ten of the authors of the manuscript for content validity: these authors are mid-career/senior pharmacists and members of the Order of Pharmacists of Lebanon Scientific Committee—Environment and Public Health Subcommittee. Several rounds of discussion using the Delphi method were conducted to reach the consensus on 31 questions. After identification of the problem area of research and defining the objectives of the questionnaire, the panel members searched the literature for previously published tools and potential items to be added to the questionnaire [8, 9, 35–38]. Tools and items were sorted and pooled in a list of items, and circulated for controlled feedback among the panel members. Items with less than 90% consensus were discussed to be kept, modified or removed from the final list. Afterward, the KAP scale was further assessed for construct validity and reliability (see the Statistical analysis section).

Sixteen questions explored participants’ knowledge of expired and unused medications, medication waste, domestic use, and risk of harmful effects), while 7 questions queried their attitude (mainly their opinion on how to solve the medication waste issue) and 8 questions investigated their practices related to the disposal of expired and unused medications. The total scores were calculated by summing up all the correct/positive answers to the knowledge, attitude, and practice questions.

### Statistical analysis

Data were analyzed on SPSS software version 25. A descriptive analysis was performed using absolute frequencies and percentages for categorical variables and means and standard deviations (SD) for quantitative measures.

Construct validity of the knowledge, attitude, and practice scales was assessed using principal component analysis (PCA). The Kaiser–Meyer–Olkin (KMO) and Bartlett’s test of sphericity were calculated to ensure the model’s adequacy. Factors with Eigenvalues > 1 were retained, and the scree plot method was used to determine the number of components to extract [39]. Moreover, the internal consistency of the studied scales was assessed using Cronbach’s alpha: internal consistency values of α ≥ 0.7 and ≥ 0.8 were considered acceptable and excellent, respectively [40]. These variables were deemed normally distributed, as verified by the visual inspection of the histogram, while the skewness and kurtosis were below |1.96|. When applicable, the normality of the scales used was verified by the normality line of the regression plot and the scatter plot of the residuals. Also, KAP scores were dichotomized into good and inappropriate with a cut-off point of 75%, where good KAP was reflected by a score of 75% and above and inappropriate KAP by a percentage strictly below 75%.

In the bivariate analysis, the independent-sample *t*-test was used to compare the means of the KAP scales between two groups, whereas the ANOVA test was used to compare three or more means. The Pearson correlation test was used to correlate continuous variables. A *p*-value less than 0.05 was considered significant.

After that, three linear regressions using the Enter method were performed, taking the KAP scales as the dependent variables. In the first linear regression, the knowledge scale was taken as the dependent variable and sociodemographic characteristics as the independent variables. In the second linear regression, the attitude scale was taken as the dependent variable, and knowledge and sociodemographic characteristics as the independent variables. In the third linear regression, the practice scale was selected as the dependent variable, and knowledge, attitude scales, and sociodemographic characteristics as the independent variables. Variables that showed a *p*-value < 0.2 in the bivariate analysis were included in the multivariable models to decrease potential confounders.

## Results

### Description of sociodemographic characteristics

The sociodemographic and other characteristics are displayed in Table 1. The mean age of the participants was 24.30 ± 9.20 years, and the mean household crowding index was 1.23 ± 0.79. Most participants were females (64.18%), single (80.1%), unemployed (66.9%), non-healthcare professionals (69.4%), had a university education level (77.4%), a low to no monthly income (60.4%), and lived in an urban city (75.0%), notably Beirut and Mount Lebanon (73.1%).

**Table 1:** Socio-demographic and other characteristics of the studied population (*N* = 735)

	Frequency	Percentage
Gender		
Female	471	64.1%
Male	264	35.9%
Education level		
Illiterate	6	0.8%
Primary	3	0.4%
Secondary	66	9.0%
University	569	77.4%
Postgraduate	91	12.4%
Region		
Beirut	388	52.8%
Mont Lebanon	149	20.3%
North	67	9.1%
South	87	11.8%
Beqaa	44	6.0%
Place of living		
Urban	551	75.0%
Rural	184	25.0%
Marital Status		
Single/divorced/widowed	589	80.1%
Married	146	19.9%
Occupation		
Unemployed	492	66.9%
Employed	243	33.1%
Monthly income		
No income	407	55.4%
Low	37	5.0%
Intermediate	135	18.4%
High	156	21.2%
Healthcare professional		
Yes	225	30.6%
No	510	69.4%
Presence of a healthcare professional in the family*		
Pharmacist	139	18.9%
Physician	87	11.8%
Nurse	117	15.9%
Dentist	54	7.3%
Other healthcare professionals	81	11.0%
	*Mean*	*SD*
Age	24.30	9.20
Household crowding index	1.23	0.79

*Each participant might have multiple answers

### Source of information about medication disposal

Table 2 describes the source of information of participants about medication disposal. More than half of the participants (56.9%) agreed that they ask the pharmacist about the storing instructions when buying new medications, and 52.8% reported getting their information about the proper disposal of expired medicines from personal readings. Only 28.3% declared never having received any information on how to dispose of unused or unwanted medications, and 44.8% admitted that they had never heard of medication waste.

**Table 2:** Source of information about medication disposal

	Frequency	Percentage
Ask the pharmacist about the storage instructions when buying new medications		
Yes	418	56.9
No	317	43.1
Ever heard of medication waste		
Yes	329	44.8
No	406	55.2
Ever received any information about how to dispose of unused or unwanted medications		
Yes	208	28.3
No	527	71.7
Get information about the proper disposal of expired medicines*		
Media	283	38.5
Physician	205	27.9
Pharmacist	362	49.3
Personal readings	388	52.8
Other	220	29.9

### Factor analysis of the KAP about medication waste scales

The knowledge scale items produced four factors with an eigenvalue over 1 accounting for a variance of 56.11% (Bartlett sphericity test *p* < 0.001; KMO = 0.823; Cronbach’s alpha = 0.784).

Regarding the attitude, three factors were yielded with a total variance of 63.18% (Bartlett test of sphericity *p* < 0.001; KMO = 0.690; Cronbach’s alpha = 0.598). The practice scale produced two factors accounting for a variance of 40.01% (Bartlett test of sphericity *p* < 0.001; KMO = 0.675; Cronbach’s alpha = 0.545) (Table 3).

**Table 3:** Factor analysis

Factor analysis of the knowledge about the disposal of expired medications					
Promax rotated matrix					
Factor	Item	Factor 1	Factor 2	Factor 3	Factor 4
1. Children are more vulnerable to the risks associated with unused/expired household medications	9	0.896			
2. Take-back programs for unused/expired medications should be mandatory	9	0.875			
3. There is a lack of adequate information on the safe disposal of unused/expired household medications	9	0.874			
4. Unused/expired medications present a potential risk at home	9	0.865			
5. Healthcare professionals provide advice on the safe disposal of unused/expired medications	9	0.625			
6. What type of medications can be flushed down the toilet? (None)	5		0.696		
7. What is the best method for medication disposal? (Ask a healthcare professional about the best way to dispose of medications)	3		0.657		
8. Does improper disposal of expired medications affect the environment and health? (Yes)	6		0.624		
9. How should medications (toxic and non-toxic) be disposed of in the garbage bin at home? (Mixed with unwanted substances then placed in a sealed container (like a zipper storage bag) and then thrown in the trash)	4		0.586		
10. Do medications reach groundwater if thrown in the toilet/sink? (Yes)	7		0.564		
11. What is the “medication take-back system” used in some countries? (Medication sharing or donation)	2		0.429		
12. Which one of the following can be considered medication waste? (Expired medications)	1			0.800	
13. Which one of the following can be considered medication waste? (Damaged medications that cannot be used)	1			0.790	
14. Which one of the following can be considered medication waste? (Once opened medications and beyond their recommended use date)	1			0.657	
15. Which one of the following can be considered medication waste? (Leftover medications)	1				0.835
16. Does the improper disposal of antibiotics lead to antimicrobial resistance? (Yes)	8				0.516
Percentage variance explained	56.11	25.30	13.32	9.97	7.50
Cronbach alpha	0.784	0.884	0.659	0.607	0.206
Kaiser–Meyer–Olkin (KMO)	0.823				
Bartlett’s test of sphericity	< 0.001				

Factor analysis of the Attitude regarding the disposal of unused and expired medications				
Promax rotated matrix				
Factor	Item	Factor 1	Factor 2	Factor 3
1. In your opinion, what are the options for reducing medication waste? (Dispense only as required)	2	0.872		
2. In your opinion, what are the options for reducing medication waste? (Give proper advice to consumer)	2	0.800		
3. Do you think that there is a need for a program to collect unused medicines from home? (Yes)	1		0.724	
4. In your opinion, what are the options for reducing medication waste? (Donate non-expired unused medications to those in need)	2		0.703	
5. In your opinion, what are the options for reducing medication waste? (Prescribe medications rationally)	2		0.677	
6. In your opinion, what are the options for reducing medication waste? (others)	2			0.872
7. In your opinion, what are the options for reducing medication waste? (Return to pharmacies)	2			0.624
Percentage variance explained	63.18	32.49	15.87	14.81
Cronbach alpha	0.598	0.679	0.556	0.301
Kaiser–Meyer–Olkin (KMO)	0.690			
Bartlett’s test of sphericity	< 0.001			

Factor analysis of the Practice regarding the disposal of unused and expired medications			
Promax rotated matrix			
Factor	Item	Factor 1	Factor 2
1. What do you do with expired medications? (Return it to the pharmacy)	5	0.763	
2. What do you do with non-expired unused medications? (Return it to the pharmacy)	4	0.703	
3. In which form do you discard medications? (I mix it with unwanted substances (such as used coffee grounds) before discarding)	6	0.575	
4. Where do you usually store your unused, leftover, or expired medications? (storage room)	2	0.439	
5. Do you check the expiry date of medications before buying them? (Yes)	1		0.842
6. Do you usually read medication disposal instructions? (Yes)	3		0.588
7. What do you do with non-expired unused medications? (Donate to charitable institutions)	4		0.401
8. Where do you usually store your unused, leftover, or expired medications? (refrigerator)	2		0.372
Percentage variance explained	40.01	25.52	14.48
Cronbach alpha	0.545	0.513	0.374
Kaiser–Meyer–Olkin (KMO)	0.675		
Bartlett’s test of sphericity	< 0.001		

### Descriptive results

The mean scores of the knowledge, attitude, and practice scales were 22.65 ± 6.20, 5.33 ± 1.51, and 3.06 ± 1.76, respectively. Considering the 75% cut-off point, 24.5%, 22.6%, and 21% of participants demonstrated good knowledge, attitude, and practice, respectively.

The majority of the participants considered expired and damaged medications medication waste (85% and 87.2%, respectively), but not leftover medications (71.6%). When questioned about medication disposal, most participants did not know about the appropriate way to dispose of it (garbage bin at home, flushing down the toilet), nor about the medication take-back system, although more than half of them (52.6%) agreed or strongly agreed that these programs should be mandatory. Also, 57.3% of participants agreed/strongly agreed that there is a lack of information regarding the safe disposal of unused/expired medications and considered that they should ask a healthcare professional about how to handle this situation. Nevertheless, they were not sure whether these professionals could provide proper advice and information (32.8% neutral). The majority were aware that improper disposal presents a potential risk at home and could affect the environment and children’s health (Table 4).

**Table 4:** Descriptive results related to Knowledge questions

	Frequency (%)
1. Which one of the following can be considered medication waste?	
*Expired medications*	
Yes*	**625 (85.0%)**
No	110 (15.0%)
*Leftover medications*	
Yes*	209 (28.4%)
**No**	**526 (71.6%)**
*Damaged medications that cannot be used*	
Yes*	**641 (87.2%)**
No	94 (12.8%)
*Once opened medications and beyond their recommended use date*	
Yes*	**532 (2.4%)**
No	203 (7.6%)
2. What is the “medication take-back system” used in some countries?	
a. Medication disposal	91 (12.4%)
b. Medication sharing or donation*	182 (24.8%)
c. **I don’t know**	**462 (62.9%)**
3. What is the best method for medication disposal?	
a. Throw it in the garbage	91 (12.4%)
b. Flush in the toilet or sink	41 (5.6%)
c. Ask a healthcare professional about the best way to dispose of medications*	**422 (57.4%)**
d. I don’t know	181 (24.6%)
4. How should medications (toxic and non-toxic) be disposed of in the garbage bin at home?	
a. As it is	98 (13.3%)
b. Crushed before discarding	75 (10.2%)
c. Mixed with unwanted substances then placed in a sealed container (like a zipper storage bag) and then thrown in the trash*	180 (24.5%)
d. **I don’t know**	**382 (52.0%)**
5. What type of medications can be flushed down the toilet?	
a. Any type of medication	62 (8.4%)
b. None*	332 (45.2%)
c. **I don’t know**	**341 (46.4%)**
6. Does improper disposal of expired medications affect the environment and health?	
a. Yes*	**503 (68.4%)**
b. No	50 (6.8%)
c. I don’t know	182 (24.8%)
7. Do medications reach groundwater if thrown in the toilet/sink?	
a. Yes*	**381 (51.8%)**
b. No	73 (9.9%)
c. I don’t know	281 (38.2%)
8. Does the improper disposal of antibiotics lead to antimicrobial resistance?	
a. Yes*	207 (28.2%)
b. No	100 (13.6%)
c. **I don’t know**	**428 (58.2%)**

Answer the following statements to the best of your knowledge					
	Strongly disagree	Disagree	Neutral	Agree	Strongly agree*
Unused/expired medications present a potential risk at home	82 (11.2%)	88 (12.0%)	197 (26.8%)	**209 (28.4%)**	**159 (21.6%)**
Children are more vulnerable to the risks associated with unused/expired household medications	64 (8.7%)	83 (11.3%)	165 (22.4%)	**184 (25.0%)**	**239 (32.5%)**
There is a lack of adequate information on the safe disposal of unused/expired household medications	86 (11.7%)	63 (8.6%)	165 (22.4%)	**180 (24.5%)**	**241 (32.8%)**
Healthcare professionals provide advice on the safe disposal of unused/expired medications	104 (14.1%)	175 (23.8%)	**241 (32.8%)**	151 (20.5%)	64 (8.7%)
Take-back programs for unused/expired medications should be mandatory	82 (11.2%)	72 (9.8%)	194 (26.4%)	**178 (24.2%)**	**209 (28.4%)**

Correct answers are marked by “*”

Numbers in bold represent the answer with the highest frequency

Most participants agreed on the need for a program to collect unused medicines from home (590; 80.3%). Several options have been suggested to reduce medication waste, including proper advice to consumers (91.3%), dispensing medication only as required (87.3%), rational prescribing (83.3%), and donating non-expired medications to those in need (79.5%) (Table 5).

**Table 5:** Descriptive results related to Attitude questions

In your opinion, what are the options for reducing medication waste?		
	Yes*	No
Dispense only as required	**642** **(87.3%)**	93 (12.7%)
Prescribe medications rationally	**612** **(83.3%)**	123 (16.7%)
Give proper advice to the consumer	**671** **(91.3%)**	64 (8.7%)
Donate non-expired unused medications to those in need	**584** **(79.5%)**	151 (20.5%)
Return to pharmacies	**497** **(67.6%)**	238 (32.4%)
Others	322 (43.8%)	**413** **(56.2%)**
Do you think that there is a need for a program to collect unused medicines from home?	**590** **(80.3%)**	145 (19.7%)

Correct answers are marked by “*”

Numbers in bold represent the answer with the highest frequency

When asked about their practice, most participants reported they do not read any instructions related to medication disposal (41.1%), and they keep non-expired unused medications at home until expiration (60.3%). The most common way of disposing of expired medication was by throwing it in household garbage (70.9%); it is usually discarded as it is (64.4%) (Table 6).

**Table 6:** Descriptive results related to practice questions

	Frequency (%)
1. Do you check the expiry date of medications before buying them?	
a. Yes*	**529** **(72.0%)**
b. No	61 (8.3%)
c. Sometimes	145 (19.7%)
2. Where do you usually store your unused, leftover, or expired medications?	
Bedroom	
Yes	213 (29.0%)
** No**	**522** **(71.0%)**
Kitchen	
Yes	251 (34.1%)
** No**	**484** **(65.9%)**
Storage room*	
Yes	293 (39.9%)
** No**	**442** **(60.1%)**
Bathroom	
Yes	66 (9.0%)
** No**	**669** **(91.0%)**
Refrigerator*	
** Yes**	**402** **(54.7%)**
No	333 (45.3%)
Car	
Yes	44 (6.0%)
** No**	**691** **(94.0%)**
Other	
Yes	222 (30.2%)
** No**	**513** **(69.8%)**
3. Do you usually read medication disposal instructions?	
a. Yes*	218 (29.7%)
b.** No**	**302** **(41.1%)**
c. Sometimes	215 (29.3%)
4. What do you do with non-expired unused medications?	
Throw away in household garbage	
Yes	214 (29.1%)
** No**	**521** **(70.9%)**
Flush in the toilet/sink	
Yes	64 (8.7%)
** No**	**671** **(91.3%)**
Keep at home until expired	
** Yes**	**443** **(60.3%)**
No	292 (39.7%)
Donate to charitable institutions*	
Yes	365 (49.7%)
** No**	**370** **(50.3%)**
Return it to the pharmacy*	
Yes	189 (25.7%)
** No**	**546** **(74.3%)**
5. What do you do with expired medications?	
Throw away in household garbage	
** Yes**	**521** **(70.9%)**
No	214 (29.1%)
Flush in the toilet/sink/	
Yes	111 (15.1%)
** No**	**624** **(84.9%)**
Burn it	
Yes	75 (10.2%)
** No**	**660** **(89.8%)**
Return it to the pharmacy*	
Yes	146 (19.9%)
No	589 (80.1%)
Use them although expired	
Yes	92 (12.5%)
No	643 (87.5%)
6. In which form do you discard medications?	
a. I dissolve medications in water before discarding	40 (5.4%)
b. **I** **discard** **medications** **as** **it** **is**	**473** **(64.4%)**
c. I mix it with unwanted substances (such as used coffee grounds) before discarding*	112 (15.2%)
d. Other	30 (4.1%)
e. I crush medications before discarding	79 (10.7%)

Correct answers are marked by “*”

Numbers in bold represent the answer with the highest frequency

### Bivariate analysis

Bivariate analyses showed that higher knowledge scores were significantly associated with the female gender, having a university education level, and having a high monthly income compared to other groups. Also, being a physician or a nurse was significantly associated with lower knowledge scores.

A higher attitude score (*r* = 0.295, *p* < 0.001) was significantly associated with higher knowledge; however, older age (*r* = − 0.085, *p* = 0.021) was related to a lower knowledge score (Table 7).

**Table 7:** Bivariate analysis taking the KAP scales as the dependent variables

	Knowledge scale	*p*-value	Attitude scale	*p*-value	Practice scale	*p*-value
	Mean ± SD		Mean ± SD		Mean ± SD	
Gender						
Female	22.99 ± 6.07	**0.045**	5.46 ± 1.41	**0.002**	2.97 ± 1.76	0.065
Male	22.03 ± 6.37		5.09 ± 1.65		3.22 ± 1.76	
Education level						
Illiterate	13.83 ± 2.56	** < 0.001**	4.00 ± 2.09	**0.023**	3.66 ± 2.80	0.202
Primary	12.66 ± 1.52		5.33 ± 2.08		3.33 ± 2.30	
Secondary	21.86 ± 5.31		4.90 ± 1.55		2.63 ± 1.79	
University	22.81 ± 6.22		5.36 ± 1.49		3.07 ± 1.75	
Postgraduate	23.09 ± 6.22		5.50 ± 1.50		3.27 ± 1.71	
Place of living						
Urban	22.89 ± 6.28	0.068	5.31 ± 1.50	0.607	3.15 ± 1.76	**0.015**
Rural	21.92 ± 5.89		5.38 ± 1.53		2.79 ± 1.73	
Marital status						
Single/divorced/widowed	22.65 ± 6.29	0.941	5.23 ± 1.55	** < 0.001**	3.08 ± 1.79	0.505
Married	22.61 ± 5.80		5.71 ± 1.24		2.97 ± 1.64	
Occupation						
Unemployed	22.82 ± 6.11	0.266	5.25 ± 1.53	**0.045**	3.01 ± 1.77	0.271
Employed	22.28 ± 6.35		5.48 ± 1.46		3.16 ± 1.74	
Monthly income						
No income	22.56 ± 6.10	**0.001**	5.18 ± 1.57	**0.010**	2.95 ± 1.70	**0.045**
Low	18.94 ± 7.01		5.16 ± 1.34		3.67 ± 1.70	
Intermediate	22.80 ± 5.93		5.48 ± 1.39		3.02 ± 1.67	
High	23.62 ± 6.19		5.62 ± 1.45		3.25 ± 1.97	
**Healthcare** professional*						
Yes	22.90 ± 6.66	0.483	5.24 ± 1.51	0.331	3.61 ± 1.83	** < 0.001**
No	22.53 ± 5.98		5.36 ± 1.51		2.82 ± 1.68	
Pharmacist						
Yes	22.97 ± 7.07	0.552	5.14 ± 1.58	0.159	3.86 ± 1.85	** < 0.001**
No	22.59 ± 6.03		5.36 ± 1.49		2.92 ± 1.71	
Physician						
Yes	19.62 ± 7.84	**0.031**	5.28 ± 1.50	0.851	3.84 ± 1.91	**0.011**
No	22.78 ± 6.08		5.33 ± 1.51		3.03 ± 1.75	
Nurse						
Yes	20.31 ± 6.41	**0.013**	4.90 ± 1.41	0.062	3.87 ± 1.96	**0.002**
No	22.78 ± 6.16		5.35 ± 1.51		3.01 ± 1.74	
Dentist						
Yes	21.73 ± 7.35	0.442	5.50 ± 1.14	0.562	4.03 ± 1.82	**0.004**
No	22.68 ± 6.15		5.32 ± 1.52		3.03 ± 1.75	
Other						
Yes	21.73 ± 6.84	0.155	5.26 ± 1.57	0.693	3.64 ± 1.75	**0.002**
No	22.76 ± 6.11		5.33 ± 1.50		2.99 ± 1.75	
Presence of a healthcare professional in the family*						
Yes	22.76 ± 6.34	0.679	5.37 ± 1.43	0.536	3.32 ± 1.86	**0.002**
No	22.57 ± 6.10		5.30 ± 1.56		2.89 ± 1.67	
Pharmacist						
Yes	21.97 ± 6.64	0.156	5.25 ± 1.44	0.496	3.44 ± 1.85	**0.005**
No	22.80 ± 6.08		5.34 ± 1.53		2.97 ± 1.73	
Physician						
Yes	21.67 ± 7.10	0.119	5.39 ± 1.57	0.693	3.68 ± 2.04	**0.003**
No	22.78 ± 6.06		5.32 ± 1.50		2.98 ± 1.70	
Nurse						
Yes	22.07 ± 6.23	0.276	5.35 ± 1.28	0.877	3.41 ± 1.78	**0.019**
No	22.75 ± 6.19		5.32 ± 1.55		3.00 ± 1.75	
Dentist						
Yes	21.46 ± 7.09	0.202	5.09 ± 1.59	0.230	3.55 ± 2.06	0.072
No	22.74 ± 6.12		5.34 ± 1.50		3.02 ± 1.73	
Other						
Yes	21.81 ± 7.23	0.266	5.30 ± 1.42	0.890	3.66 ± 1.87	**0.001**
No	22.75 ± 6.05		5.33 ± 1.52		2.99 ± 1.73	

	Correlation coefficient		Correlation coefficient		Correlation coefficient	
Age	− 0.085	**0.021**	0.076	**0.039**	− 0.051	0.171
Household crowding index	− 0.046	0.214	− 0.064	0.084	− 0.018	0.632
Knowledge scale			0.295	** < 0.001**	0.060	0.103
Attitude scale	0.295	** < 0.001**	–	**–**	0.179	** < 0.001**
Practice scale	0.060	0.103	0.179	** < 0.001**		

*Values marked in bold are significant

Considering the attitude scale as the dependent variable showed that higher attitude scores were associated with being a female vs. male, being married vs. single, being employed vs. unemployed, having a university education level, and having a high monthly income vs. other groups. Also, older age (*r* = 0.076, *p* = 0.039) and higher knowledge (*r* = 0.295, *p* < 0.001) and practice (*r* = 0.179, *p* < 0.001) scores were significantly associated with higher attitude scores (Table 7).

Taking the practice scale as the dependent variable in the bivariate analyses showed that living in an urban vs. rural area and having a high monthly income, being a healthcare professional, or having a healthcare professional in the family were significantly associated with a higher practice score. Also, a higher attitude score (*r* = 0.179, *p* < 0.001) was significantly associated with a higher practice score (Table 7).

### Multivariable analysis

A first linear regression taking the knowledge scale as the dependent variable showed that the female gender (Beta = 0.97), high monthly income (Beta = 1.68), a secondary (Beta = 6.11) or university (Beta = 6.80) education level, and postgraduate education (Beta = 7.13) were significantly associated with a higher knowledge score. However, older age (Beta = − 0.06) and a low monthly income (Beta = -3.06) were significantly associated with lower knowledge scores (Table 8, model 1).

**Table 8:** Multivariable analysis

	UB	SB	*p*-value	Confidence interval	
				Lower bound	Upper bound
*Model* *1:* *Linear* *regression* *taking* *the* *Knowledge* *total* *score* *as* *the* *dependent* *variable*					
**Gender** **(Female** **vs** Male*)	**0.979**	**0.076**	**0.039**	**0.047**	**1.911**
Education level (primary vs illiterate*)	− 0.482	− 0.005	0.911	− 8.942	7.978
**Education** **level** **(secondary** **vs** illiterate*)	**6.117**	**0.282**	**0.021**	**0.944**	**11.291**
**Education** **level** **(university** **vs.** illiterate*)	**6.801**	**0.459**	**0.008**	**1.773**	**11.829**
**Education** **level** **(postgraduate** **vs.** illiterate*)	**7.139**	**0.379**	**0.006**	**2.012**	**12.266**
Place of living (Rural vs Urban)	− 0.876	− 0.061	0.095	− 1.903	0.152
**Monthly** **income** **(low** **vs** **no** income*)	**−** **3.062**	**−** **0.108**	**0.004**	**−** **5.123**	**−** **1.001**
Monthly income (Intermediate vs no income*)	0.642	0.040	0.309	− 0.597	1.881
**Monthly** **income** **(high** **vs** **no** income*)	**1.682**	**0.111**	**0.009**	**0.420**	**2.943**
Healthcare professional in the family being a pharmacist (Yes vs No*)	− 0.417	− 0.026	0.488	− 1.599	0.764
Healthcare professional in the family is a Physician (Yes vs No*)	− 0.943	− 0.049	0.193	− 2.366	0.479
**Age**	**−** **0.061**	**−** **0.091**	**0.029**	**−** **0.116**	**−** **0.006**
**Variables** **entered** **in** **the** **model:** gender, education level, place of living, monthly income age, and having a healthcare professional in the family					
*Model* *2:* *Linear* *regression* *taking* *the* *Attitude* *total* *score* *as* *the* *dependent* *variable*					
**Gender** **(Female** **vs** Male*)	**0.369**	**0.117**	**0.001**	**0.146**	**0.592**
Education level (primary vs illiterate*)	1.338	0.056	0.187	− 0.650	3.326
Education level (secondary vs illiterate*)	0.324	0.061	0.603	− 0.897	1.544
Education level (university vs illiterate*)	0.764	0.211	0.207	− 0.424	1.952
Education level (postgraduate vs illiterate*)	0.591	0.129	0.340	− 0.625	1.808
Marital status (married vs single*)	0.284	0.075	0.124	− 0.078	0.647
Occupation (employed vs unemployed*)	0.098	0.030	0.567	− 0.237	0.432
Monthly income (low vs no income*)	0.124	0.018	0.646	− 0.407	0.655
Monthly income (intermediate vs no income*)	0.112	0.029	0.526	− 0.234	0.457
Monthly income (high vs no income*)	0.227	0.061	0.199	− 0.120	0.575
Age	0.008	0.051	0.327	− 0.008	0.025
Household crowding index	− 0.095	− 0.049	0.170	− 0.230	0.041
**Knowledge** **total** **score**	**0.069**	**0.281**	**0.000**	**0.052**	**0.086**
**Variables** **entered** **in** **the** **model:** Gender, education level, marital status, occupation level, monthly income, age, household crowding index, and knowledge score					
*Model* *3:* *Linear* *regression* *taking* *the* *Practice* *total* *score* *as* *the* *dependent* *variable*					
**Gender** **(Female** **vs** Male*)	**−** **0.329**	**−** **0.089**	**0.014**	**−** **0.590**	**−** **0.068**
**Place** **of** **living** **(Rural** **vs** Urban*)	**−** **0.372**	**−** **0.091**	**0.010**	**−** **0.655**	**−** **0.088**
Monthly income (low vs no income*)	0.571	0.071	0.053	− 0.007	1.148
Monthly income (intermediate vs no income*)	0.127	0.028	0.465	− 0.214	0.467
Monthly income (high vs no income*)	0.221	0.051	0.206	− 0.122	0.564
Age	− 0.010	− 0.050	0.205	− 0.025	0.005
**Healthcare** **professional** **(Yes** **vs** No*)	**0.722**	**0.189**	**0.000**	**0.443**	**1.001**
Presence of a healthcare professional in the family (Yes vs No*)	0.250	0.069	0.055	− 0.005	0.505
**Attitude** **total** **score**	0.571	0.071	0.053	− 0.007	1.148
Knowledge total score	0.127	0.028	0.465	− 0.214	0.467
Variables entered in the model: Gender, place of living, monthly income, age, healthcare in the family, healthcare, attitude, and knowledge scales					

*Reference group. Values marked in bold are significant

A second linear regression taking the attitude scale as the dependent variable showed that the female gender (Beta = 0.36) and a higher knowledge score (Beta = 0.06) were significantly associated with a more positive attitude regarding the disposal of unused or expired medications (Table 8, model 2).

A third linear regression taking the practice scale as the dependent variable showed that being a healthcare professional (Beta = 0.72) was significantly associated with a higher practice score. However, being a female (Beta = − 0.32) and living in a rural area (Beta = − 0.37) were significantly associated with lower practice scores (Table 8, model 3).

When considering the different categories of healthcare professionals or having a healthcare professional in the family, none of the categories were associated with the total practice score (*p* > 0.05 for all) (Additional file 2).

## Discussion

This study first aimed to validate a questionnaire assessing KAP toward unused or expired medications. To the best of our knowledge, it is the first to perform such a validation in Lebanon. Other studies have developed and validated questionnaires related to KAP regarding unused medications at home (QUM-Qatar [33] or ReDiUM in Malaysia [36]). In our study, using the Promax rotation, the PCA showed good internal consistency (IC) for the knowledge scale (Cronbach’s alpha of 0.784), similar to what was reported in the Malaysian study (overall Cronbach’s alpha value was 0.727) [36]. However, the IC values for the attitude and practice scores were lower than those reported in the Qatari study [33] (0.598 and 0.545 versus 0.82 and 0.84, respectively), suggesting the need to improve the current questionnaire for a better assessment of appropriate disposal measures in future studies.

Our study also assessed correlates of the KAP scales, which would allow for implementing targeted interventions at the national level. Our results highlighted poor knowledge of the general population of medication disposal (only 24.5% had good knowledge according to our score calculations). Most participants admitted having a lack of adequate information and clear instructions regarding the best disposal method, similar to previous findings in several other countries, such as Saudi Arabia, New Zealand, Bangladesh, Malta, and Ireland [12, 22, 41].

However, a higher perceived knowledge score was noted in participants with intermediate and higher education levels (secondary, university, or postgraduate versus illiterate) and higher incomes, as published elsewhere [42]. A possible explanation could be that people with higher levels of education and more stable financial conditions tend to seek information more intuitively than others and have a better grasp of their surroundings. Conversely, similar to other researchers’ results, less educated participants are more likely to experience difficulties seeking new information and might not be aware of the consequences of improperly disposing of medicines [43].

Knowledge and attitudes were significantly associated with gender, with females scoring significantly higher on both. Results related to attitude are consistent with previous findings from Saudi Arabia, showing that females were significantly more willing to use medication collection facilities than males and considered having more individual responsibility for appropriate medication disposal [41, 44].

As for practice, the most common method used for expired medication disposal was household garbage, in line with the results of a 10-year literature review (2005–2015), including Kuwait, Qatar, Saudi Arabia, the United Kingdom, and India, among others [45]. Furthermore, our study revealed that most participants did not read any instructions related to medication disposal, and they kept non-expired unused medications at home until their expiration. This finding is particularly alarming in a country such as Lebanon, where most medications, including some antibiotics, can be accessed without a prescription, mainly in lower socioeconomic communities [46]. Also, both the current steep economic crisis that resulted in the local currency devaluation and the COVID-19 pandemic compelled people to hoard medications at home, thus anticipating the lifting of subsidies on some pharmaceuticals by the central bank [47, 48]. One could expect that a substantial amount of these medications might not be used and would therefore end up being thrown in the garbage. This harmful practice should be controlled, and the relevant authorities are urged to promote other disposal methods, such as medication take-back sites, where consumers can drop off expired or unwanted medications.

Interestingly, participants believed they should ask healthcare professionals about the proper disposal methods, but were skeptical about their ability to provide adequate information. Nevertheless, multivariable analyses have shown that all healthcare professionals, including pharmacists, physicians, dentists, and nurses, had significantly higher practice scores than non-healthcare professionals. This result is not surprising since healthcare workers are the key personnel responsible for medication and medical waste management [49].

### Implications for practice

Taken together, our results emphasize the need for a national waste management strategy that defines the roles of healthcare professionals, governmental and non-governmental organizations, and the pharmaceutical industry. Educational campaigns offered by different parties, including media, schools, pharmacists, and other healthcare professionals, can raise awareness about the environmental problems stemming from pharmaceutical residues and the proper measures and disposal standards to reduce medication waste. [12, 49, 50]

### Limitations and strengths

Our study has some limitations. Its cross-sectional design does not allow for causal inference. Although the sample size was sufficient for statistical analyses to be carried out, the results could have been more representative with a larger sample. Another limitation was that participants were recruited using an online questionnaire; most of them were young, lived in urban areas, had a high level of education, and had good computer literacy. Hence, our findings might not be generalizable to the entire population [45]. Furthermore, some participants may not have admitted to inappropriate medication disposal to please researchers, leading to social desirability bias or even recall bias. Nevertheless, this bias was minimized by assuring the participants of the study anonymity and the importance of their frankness. Despite all these limitations, our study is still among the very few in the region to have validated a KAP questionnaire about the disposal of unused or expired medications for construct validity and reliability.

## Conclusion

This study revealed relatively low levels of knowledge, attitude, and practice related to medication disposal in Lebanon. Participants admitted having a lack of information and agreed on the need for specific programs for medication waste management. Several factors were shown to be associated with higher KAP scores in various aspects of medication disposal, including gender, age, education level, and profession (healthcare professionals), suggesting the need to consider those when implementing targeted corrective measures. This study could be the ground for a medication waste management national strategy in Lebanon. From a research perspective, it showed the need to develop a more comprehensive questionnaire to have better insight into KAP regarding the disposal of unused and expired medication among the general population and to expand it at a later stage to evaluate biomedical waste management among healthcare professionals.

## Supplementary Information

**Additional** **file** **1**. Questionnaire about knowledge, attitudes and Practice toward the disposal of expired medications among the Lebanese General Population.

**Additional** **file** **2**. Multivariable analysis taking the Practice total score as the dependent variable (being member of the different healthcare professions or having a family member as a healthcare professional as independent variables)

## Data Availability

The datasets used and/or analyzed during the current study are available from the corresponding author on reasonable request.

## Abbreviations

US: United States
SDG: Sustainable development goals
DG: Development goals
FDA: Food and Drug Administration
MOPH: Ministry of Public Health
LPIA: Lebanese Pharmaceutical Importers and Wholesalers Association
KAP: Knowledge, attitude, practices
SPSS: Statistical Package for the Social Sciences
SD: Standard deviations
PCA: Principal component analysis
KMO: Kaiser–Meyer–Olkin
