# Supplementary material for: Assessment of knowledge, attitude, and practice regarding the disposal of expired and unused medications among the Lebanese population
Source: J Pharm Policy Pract. 2022 Dec 30;15:107. doi: 10.1186/s40545-022-00506-z (PMC9802024; doi:10.1186/s40545-022-00506-z)
Supplement: Supplementary file 1 — Additional file 1. Questionnaire about knowledge, attitudes and Practice toward the disposal of expired medications among the Lebanese General Population. [file 40545_2022_506_MOESM1_ESM.pdf]

Knowledge, Attitudes, and Practice Toward the Disposal of Expired Medications Among the Lebanese General Population/المعرفة والمواقف والممارسة تجاه التخلص من الأدوية منتهية الصلاحية بين اللبنانيين

Dear participant,

You are invited to participate in a survey about knowledge, attitude, and practice towards the disposal of expired medications conducted by the Scientific Committee at the Order of Pharmacists of Lebanon.
Your participation in this study is voluntary and anonymous, and the information gathered in this 10-minute questionnaire will be treated confidentially.
By completing it, you are consenting to participate in this study.

We thank you in advance for your time,

The OPL Scientific Committee.

عزيزي المشارك،

أنت مدعو للمشاركة في استطلاع حول المعرفة، والسلوك، والممارسة تجاه التخلص من الأدوية منتهية الصلاحية ، والتي تجريها اللجنة العلمية في نقابة الصيادلة في لبنان
إن مشاركتك في هذه الدراسة طوعية وجميع المعلومات التي سيتم جمعها غير اسمية وسيتم التعامل معها بسرية تامة.
إن إكمال هذا الاستبيان يستغرق حوالي 10 دقائق ويشير الى الموافقة على المشاركة.

شكراً سلفاً على وقتك،

*Obligatoire

Informed Consent/موافقة مسبقة

1. Please check all the boxes to proceed to the survey/الرجاء اختيار جميع المربعات أدناه لبدء الاستبيان *

Plusieurs réponses possibles.

- [ ] I have read and understood the above information/لقد قرأت وفهمت المعلومات الواردة أعلاه
- [ ] I understand that my participation is voluntary/أدرك أن مشاركتي طوعية
- [ ] I understand that my data will be kept confidential/أدرك أن المعلومات التي سأقدمها ستبقى سرية
- [ ] I agree to participate in this study/أوافق على المشاركة في البحث

Sociodemographic characteristics/الخصائص الاجتماعية الديموغرافية

2. 1. Age/العمر *

____

3. 2. Gender/الجنس *

Une seule réponse possible.

( ) Male/ذكر
( ) Female/أنثى

4. 3. Education level/مستوى التعليم *

Une seule réponse possible.

( ) Illiterate/أمي
( ) Primary/إبتدائي
( ) Secondary/ثانوي
( ) University (Bachelor's degree)/جامعي
( ) Postgraduate degree (Master’s, PhD)/شهادة عليا

5. 4. Place of Living/مكان الإقامة *

Une seule réponse possible.

- Beirut/بيروت
- Mount Lebanon/جبل لبنان
- North/الشمال
- South/الجنوب
- Beqaa/البقاع

6. 5. Place of living type/نوع مكان الإقامة *

Une seule réponse possible.

- Urban/مدينة
- Rural/ريف

7. 6. Number of persons living in the same household, including you/عدد الأشخاص الذين يعيشون في نفس المنزل، بما فيهم أنت *

____

8. 7. Number of rooms in your house, excluding the kitchen and bathrooms/عدد الغرف في منزلك ماعدا المطبخ والحمامات *

____

9. 8. Marital status/الحالة الاجتماعية *

Une seule réponse possible.

- Single/أعزب
- Married/متزوج
- Divorced/مطلق
- Widowed/أرمل

10. 9. Occupation/المهنة *

Une seule réponse possible.

- Self-Employed/اعمل لحسابي الخاص
- Employed (private sector)/موظف قطاع خاص
- Employed (public sector)/موظف قطاع عام
- I do not work (student, housewife, retired, I lost my job)/لا أعمل (طالب، ربة منزل، متقاعد، فقدت وظيفتي)

11. 10. Monthly income/الدخل الشهري *

Une seule réponse possible.

- No income/لا دخل
- <675,000 LBP
- 675,000-1,500,000 LBP
- 1,500,000-3,000,000 LBP
- >3,000,000 LBP

12. 11. Are you a healthcare professional?/هل أنت متخصص في الرعاية الصحية؟ *

Une seule réponse possible par ligne.

	Yes/نعم	No/لا
**Pharmacist/صيدلاني**		
**Doctor/طبيب**		
**Nurse/ممرض**		
**Dentist/طبيب اسنان**		
**Other healthcare professional/أخصائي رعاية صحية آخر**		

13. 12. Is there a healthcare professional at home?/هل يوجد أخصائي رعاية صحية في منزلك؟ *

Une seule réponse possible par ligne.

	Yes/نعم	No/لا
**Pharmacist/صيدلاني**		
**Doctor/طبيب**		
**Nurse/ممرض**		
**Dentist/طبيب اسنان**		
**Other healthcare professional/أخصائي رعاية صحية آخر**		

Knowledge about the disposal of expired medications/المعرفة حول كيفية التخلص من الأدوية منتهية الصلاحية

14. 13. Do you usually ask the pharmacist about the storing instructions when buying new medications?/هل تسأل الصيدلي عادة عن تعليمات التخزين عند شراء الأدوية الجديدة؟ *

Une seule réponse possible.

- Yes/نعم
- No/لا

15. 14. Have you ever heard of medication waste?/هل سمعت من قبل عن نفايات الأدوية؟ *

Une seule réponse possible.

- Yes/نعم
- No/لا

16. 15. Have you ever received any information about how to dispose of unused or unwanted medications?/هل تلقيت يومًا أي معلومات حول كيفية التخلص من الأدوية غير المستخدمة أو غير المرغوب فيها؟ *

Une seule réponse possible.

- Yes/نعم
- No/لا

17. 16. Where do you get your information about the proper disposal of expired medicines?/من أين تحصل على معلوماتك حول التخلص السليم من الأدوية منتهية الصلاحية؟ *

Une seule réponse possible par ligne.

	Yes/نعم	No/لا
**Media/وسائل الإعلام**		
**Physician/الطبيب المعالج**		
**Pharmacist/الصيدلاني**		
**Personal readings/ قراءات شخصية**		
**Other/غير ذلك**		

18. 17. Which one of the following can be considered medication waste?/أي مما يلي يمكن اعتباره نفايات أدوية؟ *

Une seule réponse possible par ligne.

	Yes/نعم	No/لا
**Expired medications/ الأدوية منتهية الصلاحية**	⬭	⬭
**Leftover medications/ الأدوية المتبقية**	⬭	⬭
**Damaged medications that cannot be used/الأدوية التالفة التي لا يمكن استخدامها**	⬭	⬭
**Once opened medications and beyond their recommended use date/الأدوية المفتوحة وبعد تاريخ الاستخدام الموصى به بعد فتحها**	⬭	⬭
**Row 5**	⬭	⬭

19. 18. What is the “medication take-back system” used in some countries?/ما هو "نظام استرجاع الأدوية" المستخدم في بعض البلدان؟ *

Une seule réponse possible.

- ⬭ Medication disposal/التخلص من الأدوية
- ⬭ Medication sharing or donation/تقاسم الأدوية أو التبرع
- ⬭ I don’t know/لا أعرف

20. 19. What is the best method for medication disposal?/ما هي أفضل طريقة للتخلص من الأدوية؟ *

Une seule réponse possible.

- Throw in the garbage/رميها في القمامة
- Flush in the toilet or sink/رميها في المرحاض أو المغسلة
- Ask a healthcare professional about the best way to dispose of medications/اسأل أخصائي رعاية صحية عن أفضل طريقة للتخلص من الأدوية
- I don't know/لا أعرف

21. 20. How should medications (toxic and non-toxic) be disposed of in the garbage bin at home?/كيف يجب التخلص من الأدوية (السامة وغير السامة) في سلة المهملات بالمنزل؟ *

Une seule réponse possible.

- As it is/كما هي
- Crushed before discarding/تسحق قبل رميها
- Mixed with unwanted substances (such as used coffee grounds), then placed in a sealed container (like zipper storage bag) and then thrown in the trash/تخلط مع مواد غير مرغوب فيها (مثل تفل القهوة)، ثم توضع في حاوية مغلقة (مثل كيس تخزين بسحاب) ثم ترمى في سلة المهملات
- I don't know/لا أعرف

22. 21. What type of medications can be flushed down the toilet?ما نوع الأدوية التي يمكن رميها في المرحاض؟ *

Une seule réponse possible.

- Any type of medication/أي نوع من الأدوية
- None/لا يمكن رمي أي من الأدوية في المرحاض
- I don't know/لا أعرف

23. 22. Does improper disposal of expired medications affect the environment and health? هل التخلص غير السليم من الأدوية منتهية الصلاحية يؤثر على البيئة والصحة؟ *

Une seule réponse possible.

- Yes/نعم
- No/لا
- I don’t know/لا أعرف

24. 23. Do medications reach groundwater if thrown in the toilet/sink?هل تصل الأدوية إلى المياه الجوفية إذا ألقيت في المرحاض أو المغسلة؟ *

Une seule réponse possible.

- Yes/نعم
- No/لا
- I don’t know/لا أعرف

25. 24. Does the improper disposal of antibiotics lead to antimicrobial resistance?/هل التخلص غير السليم من المضادات الحيوية يؤدي إلى مقاومة المضادات الحيوية؟ *

Une seule réponse possible.

- Yes/نعم
- No/لا
- I don’t know/لا أعرف

26. 25. Answer the following statements to the best of your knowledge/أجب على العبارات التالية بأفضل ما لديك من معلومات *

Une seule réponse possible par ligne.

	Strongly disagree/لا أوافق بشدة	Disagree/ لا أوافق	Neutral/ محايد	Agree/ أوافق	Strongly agree/أوافق بشدة
**Unused/expired medications present a potential risk at home/ الأدوية غير المستخدمة/منتهية الصلاحية تشكل خطرًا محتملاً في المنزل**	◯	◯	◯	◯	◯
**Children are more vulnerable to the risks associated with unused/expired household medications/ الأطفال أكثر عرضة للمخاطر المرتبطة بالأدوية غير المستخدمة/منتهية الصلاحية في المنزل**	◯	◯	◯	◯	◯
**There is lack of adequate information on safe disposal of unused/expired household medications/ هناك نقص في المعلومات الكافية حول التخلص الآمن من الأدوية المنزلية غير المستخدمة أو منتهية الصلاحية**	◯	◯	◯	◯	◯
**Healthcare**	◯	◯	◯	◯	◯

**professionals provide advice on the safe disposal of unused/expired medications/ يقدم أخصائيو الرعاية الصحية النصائح بشأن التخلص الآمن من الأدوية غير المستخدمة/منتهية الصلاحية**					
**Take-back programs of unused/expired medications should be mandatory/يجب أن تكون برامج استعادة الأدوية غير المستخدمة/منتهية الصلاحية إلزامية**					

27. 26. How could hazardous effects of expired medications be minimized or controlled?/كيف يمكن التقليل من الآثار الخطيرة للأدوية منتهية الصلاحية أو التحكم فيها؟ *

Une seule réponse possible par ligne.

	Yes/نعم	No/لا
**Provide proper guidance to the consumer/ تقديم التوجيهات المناسبة للمستهلك**	◯	◯
**Prescribe the adequate quantities and durations that ensure patient compliance/ وصف الكميات والمدة المناسبة التي تضمن التزام المريض بالعلاج**	◯	◯
**Reduce the number of prescribed medications per prescription/ تقليل عدد الأدوية الموصوفة في كل وصفة طبية**	◯	◯
**Donate to or share unused medications to friends or relatives before it gets expired/ تبرع بالأدوية غير المستخدمة أو شاركها مع الأصدقاء أو الأقارب قبل انتهاء صلاحيتها**	◯	◯
**Keep expired medications in a safe place/ احتفظ بالأدوية منتهية الصلاحية في مكان آمن**	◯	◯
**Dispose of expired medications in the toilet/ تخلص من الأدوية منتهية الصلاحية في المرحاض**	◯	◯
**Burn expired medications/ أحرق الأدوية منتهية الصلاحية**	◯	◯

**None of the above/ لا شيء مما ورد أعلاه**	⬭	⬭

Attitude regarding the disposal of unused and expired medications/الموقف من التخلص من الأدوية غير المستخدمة والمنتهية الصلاحية

28. 27. Do you think that there is a need for a program to collect unused medicines from home?/هل تعتقد أن هناك حاجة لبرنامج لجمع الأدوية غير المستخدمة من المنزل؟ *

Une seule réponse possible.

- Yes/نعم
- No/لا
- I don't know/لا أعرف

29. 28. Who do you think is responsible for creating public awareness about the proper disposal of unused and expired medications?/ من برأيك المسؤول عن خلق وعي عام حول التخلص السليم من الأدوية غير المستخدمة والمنتهية الصلاحية؟ *

Une seule réponse possible par ligne.

	Yes/نعم	No/لا
**Media/وسائل الإعلام**	⬭	⬭
**Physician/الطبيب المعالج**	⬭	⬭
**Pharmacist/الصيدلاني**	⬭	⬭
**Pharmaceutical companies and manufacturers/ شركات الأدوية والمصنعين**	⬭	⬭
**Competent authorities/ السلطات المختصة**	⬭	⬭

30. 29. In your opinion, what measures can improve awareness of consumers regarding the safe disposal of medications?/ في رأيك، ما هي الإجراءات التي من شأنها تحسين وعي المستهلكين بشأن التخلص الآمن من الأدوية؟ *

Une seule réponse possible par ligne.

	Yes/نعم	No/لا
**Patient education by pharmacists, doctors, or nurses/ تثقيف المريض من قبل الصيادلة أو الأطباء أو الممرضات**		
**Information in media/ معلومات في وسائل الإعلام**		
**Awareness programs by the government/ برامج توعوية من قبل الحكومة**		
**Written instructions on medication boxes/ تعليمات مكتوبة على علب الأدوية**		

31. 30. In your opinion, what are the options for reducing medication waste?/ ما هي برأيك خيارات الحد من نفايات الأدوية؟ *

Une seule réponse possible par ligne.

	Yes/نعم	No/لا
**Dispense only as required/ صرف الدواء فقط كما هو مطلوب**	⬭	⬭
**Prescribe medications rationally/ ترشيد وصف الأدوية**	⬭	⬭
**Give proper advices to consumer/ إعطاء النصائح المناسبة للمستهلك**	⬭	⬭
**Donate non-expired unused medications to those in need/ التبرع للمحتاجين بالأدوية غير منتهية الصلاحية وغير المستخدمة**	⬭	⬭
**Return to pharmacies/ إعادة الأدوية الى الصيدليات**	⬭	⬭
**Other/ غير ذلك**	⬭	⬭

Practice regarding the disposal of unused and expired medications/الممارسة المتعلقة بالتخلص من الأدوية غير المستخدمة والمنتهية الصلاحية

32. 31. How do you buy medications?/كيف تشتري الأدوية؟ *

Une seule réponse possible par ligne.

	Yes/نعم	No/لا
**Upon prescription/ بناء على وصفة طبية**		
**Over the counter/دون وصفة طبية**		
**On the advice of the pharmacist/بناء على نصيحة الصيدلي**		
**From or on the advice of friends or colleagues/من أو بناءً على نصيحة الأصدقاء أو الزملاء**		

33. 32. Types of medication stored at home/أنواع الأدوية المخزنة في المنزل *

Une seule réponse possible par ligne.

	Yes/نعم	No/لا
**Analgesics/المسكنات**	⬭	⬭
**Antibiotics/المضادات الحيوية**	⬭	⬭
**Chronic disease medications/أدوية الأمراض المزمنة**	⬭	⬭
**Vitamins and food supplements/الفيتامينات والمتممات الغذائية**	⬭	⬭
**Syrups/شراب**	⬭	⬭
**Topical medications (creams, ointments, and solutions)/الأدوية الموضعية (الكريمات والمراهم والمحاليل)**	⬭	⬭
**Other medications/ أدوية أخرى**	⬭	⬭

34. 33. Do you check the expiry date of medications before buying them?/هل تتحقق من تاريخ انتهاء صلاحية الأدوية قبل شرائها؟ *

Une seule réponse possible.

- ⬭ Yes/نعم
- ⬭ No/لا
- ⬭ Sometimes/أحياناً

35. 34. Where do you usually store your unused, leftover, or expired medications?/أين تخزن عادة الأدوية غير المستخدمة أو المتبقية أو منتهية الصلاحية؟ *

Une seule réponse possible par ligne.

	Yes/نعم	No/لا
**Bedroom/غرفة النوم**	⬭	⬭
**Kitchen/المطبخ**	⬭	⬭
**Storage room/غرفة التخزين**	⬭	⬭
**Bathroom/الحمام**	⬭	⬭
**Refrigerator/البراد**	⬭	⬭
**Car/السيارة**	⬭	⬭
**Other/غير ذلك**	⬭	⬭

36. 35. Do you usually read medication disposal instructions?/هل تقرأ عادة تعليمات التخلص من الأدوية؟ *

Une seule réponse possible.

- ⬭ Yes/نعم
- ⬭ No/لا
- ⬭ Sometimes/أحيانا

37. 36. Specify the number of boxes of unused medications at home/حدد عدد علب الأدوية غير المستخدمة في المنزل *

Une seule réponse possible.

- ( ) 0
- ( ) 1-5
- ( ) 6-10
- ( ) >10

38. 37. What is the main reason for the presence of some unused medications at your place?/ما هو السبب الرئيسي لوجود بعض الأدوية غير المستخدمة في منزلك؟ *

Une seule réponse possible.

- ( ) Disease or condition resolved/تم الشفاء من المرض أو الحالة
- ( ) Medication stopped because of side effects/توقيف الدواء بسبب الآثار الجانبية
- ( ) Medication change/تغيير الدواء
- ( ) Autre : ____________________

39. 38. What do you do with non-expired unused medications?/ماذا تفعل بالأدوية غير المنتهية الصلاحية وغير المستخدمة؟ *

Une seule réponse possible par ligne.

	Yes/نعم	No/لا
**Throw away in household garbage/ ارميها في القمامة المنزلية**		
**Flush in the toilet/sink/ارميها في المرحاض أو المغسلة**		
**Keep at home until expired/احتفظ بها في المنزل حتى تنتهي صلاحيتها**		
**Donate to charitable institutions/أتبرع بها لمؤسسات خيرية**		
**Return it to the pharmacy/أعيدها إلى الصيدلية**		

40. 39. What do you do with expired medications?/ماذا تفعل بالأدوية منتهية الصلاحية؟ *

Une seule réponse possible par ligne.

	Yes/نعم	No/لا
**Throw away in household garbage/ ارميها في القمامة المنزلية**	◯	◯
**Flush in the toilet/sink/ارميها في المرحاض أو المغسلة**	◯	◯
**Burn it/أحرقها**	◯	◯
**Return it to the pharmacy/أعيدها إلى الصيدلية**	◯	◯
**Use them although expired/استخدمها على الرغم من انتهاء صلاحيتها**	◯	◯

41. 40. In which form do you discard medications?/في أي شكل تتخلص من الأدوية؟ *

Une seule réponse possible.

- ◯ I crush medications before discarding/أسحق الأدوية قبل التخلص منها
- ◯ I dissolve medications in water before discarding/أذوب الأدوية بالماء قبل التخلص منها
- ◯ I discard medications as it is/أتخلص من الأدوية كما هي
- ◯ I mix it with unwanted substances (such as used coffee grounds) before discarding/ أخلطها مع مواد غير مرغوب فيها (مثل تفل القهوة) قبل التخلص منها
- ◯ Autre : ____________

Ce contenu n'est ni rédigé, ni cautionné par Google.

Google Forms
