# Supplementary material for: Assessment of knowledge, attitude, and practice regarding the disposal of expired and unused medications among the Lebanese population
Source: J Pharm Policy Pract. 2022 Dec 30;15:107. doi: 10.1186/s40545-022-00506-z (PMC9802024; doi:10.1186/s40545-022-00506-z)
Supplement: Supplementary file 2 — Additional file 2. Multivariable analysis taking the Practice total score as the dependent variable (being member of the different healthcare professions or having a family member as a healthcare professional as independent variables) [file 40545_2022_506_MOESM2_ESM.docx]

**Additional File 2:** Multivariable analysis taking the Practice total score as the dependent variable (being member of the different healthcare professions or having a family member as a healthcare professional as independent variables).

| **Model 1: Linear regression taking the Practice total score as the dependent variable.** | | | | | |
| --- | --- | --- | --- | --- | --- |
|  | **UB** | **SB** | **p-value** | **Confidence interval** | |
|  |  |  |  | **Lower Bound** | **Upper Bound** |
| **Gender (Female vs Male*)** | **-.301** | **-.082** | **.027** | **-.568** | **-.034** |
| **Place of living (Rural vs Urban*)** | **-.321** | **-.079** | **.029** | **-.610** | **-.033** |
| Monthly income (low vs no income*) | .491 | .061 | .106 | -.105 | 1.088 |
| Monthly income (Intermediate vs no income*) | .096 | .021 | .586 | -.250 | .443 |
| Monthly income (High vs no income*) | .273 | .063 | .125 | -.076 | .621 |
| Age | **-.016** | **-.082** | **.039** | **-.031** | **-.001** |
| **Healthcare professional being a doctor (Yes vs No*)** | .250 | .029 | .452 | -.402 | .903 |
| **Healthcare professional being a Nurse (Yes vs No*)** | .355 | .046 | .260 | -.263 | .974 |
| **Healthcare professional other type (Yes vs No*)** | .316 | .056 | .172 | -.138 | .769 |
| **Presence of a healthcare in the family pharmacist (Yes vs No*)** | .207 | .046 | .238 | -.137 | .551 |
| **Presence of a healthcare in the family doctor (Yes vs No*)** | .359 | .066 | .115 | -.087 | .806 |
| **Presence of a healthcare in the family Nurse (Yes vs No*)** | .066 | .014 | .731 | -.310 | .441 |
| **Presence of a healthcare in the family dentist (Yes vs No*)** | -.053 | -.008 | .845 | -.589 | .482 |
| **Presence of a healthcare in the family other (Yes vs No*)** | .282 | .050 | .223 | -.172 | .736 |
| **Attitude total score** | **.220** | **.189** | **.000** | **.133** | **.307** |
| Knowledge total score | .006 | .020 | .600 | -.016 | .027 |
| Variables entered in the model: Gender, place of living, monthly income, age, being a Healthcare professional: doctor, Nurse, other type of healthcare professional, Presence of a healthcare in the family: pharmacist, doctor, Nurse, dentist and other healthcare professional, attitude and knowledge scales. | | | | | |
| ***Reference group** | | | | | |
